# Morphing of Ibogaine: A Successful Attempt into the Search for Sigma-2 Receptor Ligands

**DOI:** 10.3390/ijms20030488

**Published:** 2019-01-23

**Authors:** Giuseppe Floresta, Maria Dichiara, Davide Gentile, Orazio Prezzavento, Agostino Marrazzo, Antonio Rescifina, Emanuele Amata

**Affiliations:** 1 Department of Drug Sciences, University of Catania, V.le A. Doria, 95125 Catania, Italy; giuseppe.floresta@unict.it (G.F.); maria.dichiar@unict.it (M.D.); davide.gentile@studium.unict.it (D.G.); prezzave@unict.it (O.P.); marrazzo@unict.it (A.M.); 2 Consorzio Interuniversitario Nazionale di ricerca in Metodologie e Processi Innovativi di Sintesi (C.I.N.M.P.S.), Via E. Orabona, 4, 70125 Bari, Italy

**Keywords:** sigma-2 receptor, TMEM97, scaffold-hopping, molecular docking, Ibogaine, Pinoline, Incazane

## Abstract

Ibogaine is a psychoactive indole alkaloid with high affinity for several targets including the *σ*_2_ receptor. Indeed, extensive data support the involvement of the *σ*_2_ receptor in neurological disorders, including Alzheimer’s disease, schizophrenia, alcohol abuse and pain. Due to its serious side effects which prevent ibogaine from potential clinical applications, novel ibogaine derivatives endowed with improved *σ*_2_ receptor affinity may be particularly beneficial. With the purpose to facilitate the investigation of iboga alkaloid derivatives which may serve as templates for the design of selective *σ*_2_ receptor ligands, here we report a deconstruction study on the ibogaine tricyclic moiety and a successive scaffold-hopping of the indole counterpart. A 3D-QSAR model has been applied to predict the *σ*_2_ p*K*_i_ values of the new compounds, whereas a molecular docking study conducted upon the *σ*_2_ receptor built by homology modeling was used to further validate the best-scored molecules. We eventually evaluated pinoline, a carboline derivative, for *σ*_2_ receptor affinity through radioligand binding assay and the results confirmed the predicted high µM range of affinity and good selectivity. The obtained results could be helpful in the drug design process of new ibogaine simplified analogs with improved *σ*_2_ receptor binding capabilities.

## 1. Introduction

First introduced as subtypes of the opioid receptor and as high-affinity phencyclidine binding sites, sigma receptors are now recognized as a particular and unique receptor class. Two subtypes are currently known, denoted as sigma-1 (*σ*_1_) and sigma-2 (*σ*_2_) receptors, having a different structures, biological functions, and pharmacological profiles.

Sigma-1 *σ*_1_ receptor has been identified as a 25.3 kDa chaperone protein within the mitochondria-associated endoplasmic reticulum membranes (MAMs) [1]. Recently, the crystal structure of the human *σ*_1_ receptor has been reported revealing a trimeric architecture [2]. Sigma-1 *σ*_1_ receptor is highly expressed in both the central and peripheral nervous system, with involvement in the production of neurotrophic factors and in the protection of the mitochondrial integrity [3,4]. In this view, *σ*_1_ receptor agonists represent potential therapeutic agents for the treatment of several neuropsychiatric and neurodegenerative disorders, whereas *σ*_1_ receptor antagonists have been reported for their antiproliferative and antiangiogenic effects, in addition to the modulation of pain and drug abuse-related conditions [5,6,7,8,9,10].

Sigma-2 *σ*_2_ receptor is a poorly understood protein whose identification has been controversial. For a long time its binding site has been postulated to be located in the progesterone receptor membrane component 1 (PGRMC1). A recent study has highlighted that *σ*_2_ receptor and PGRMC1 are different proteins since the presence or absence of PGRMC1 has no impact on *σ*_2_ ligands binding ability [11]. In 2017, Alon and coworkers [12] identified the *σ*_2_ receptor as an endoplasmic reticulum-resident transmembrane protein (TMEM97) playing a role in the cholesterol homeostasis and the sterol transporter Niemann–Pick disease type C1. Despite the challenges in identifying its true identity, *σ*_2_ receptor has earned a growing scientific interest due to its involvement in several disease states. High levels of the *σ*_2_ receptor have been found in several cancer cells and proliferating tumors such as lung, colorectal, ovarian, and breast cancers [13]. Extensive data support the utility of sigma-receptor ligands as cancer therapeutics and diagnostic tools [14,15]. Due to a 10-fold higher density in proliferating tumor cells than in quiescent tumor cells, *σ*_2_ receptor also represents an important clinical biomarker for determining the proliferative status of solid tumors [14]. The fluorine (F18) ISO-1 is a promising positron emission tomography (PET) ligand evaluated in clinical trials for the imaging of *σ*_2_ receptor binding in primary breast cancer [16].

More recently, *σ*_2_ receptor has been implicated in neurological disorders, including Alzheimer’s disease, schizophrenia, alcohol abuse, and pain [17,18,19,20]. The small-molecule CT1812, a *σ*_2_ receptor antagonist whose structure has not been disclosed, is currently under clinical trial in patients with mild to moderate Alzheimer’s disease [21]. Also, Roluperidone (MIN-101) is in phase III clinical trials for the treatment of negative symptoms of schizophrenia [21].

**Figure 1:**
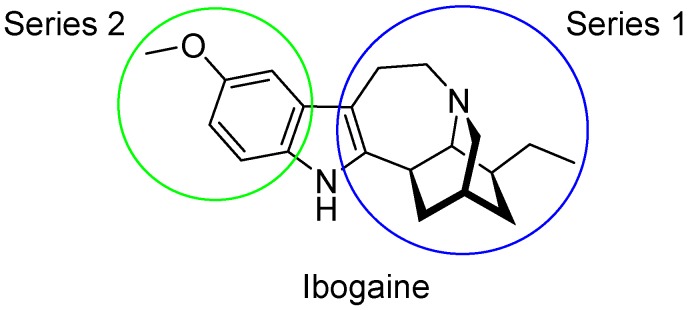
Series 1 and 2 derived from ibogaine.

Ibogaine (Figure 1), a psychoactive indole alkaloid, is a typical “dirty drug” with high affinity for a panel of targets including NMDA, *κ*- and *µ*-opioid receptors and *σ*_2_ receptor sites [22]. Ibogaine has also shown to interact with the acetylcholine, serotonin, and dopamine systems and to modify the expression of some proteins including substance P and brain-derived neurotrophic factor (BDNF) [23,24]. Initially used for its hallucinogenic properties, ibogaine has been then investigated for its potential in treating drug abuse [25]. However, little research in humans has been done due to the severe side effects and death following its ingestion, including tremors, neurotoxicity, and cardiotoxicity [26].

As mentioned above, the receptor sites through which ibogaine mediates its effects are not known with certainty even if clear evidence indicates that ibogaine and other iboga alkaloids interact with *σ*_2_ receptors. Indeed, ibogaine shows a moderate nanomolar affinity for *σ*_2_ receptor with a *K*_i_ value of 201 nM and good selectivity over *σ*_1_ site (*K*_i_ 8554 nM) [22]. Lower affinity values for other neurotransmitter receptors have been showed [27,28,29]. Similar *K*_i_ values have been reported for other iboga alkaloid analogs in which the presence or the position of the methoxy group on the aromatic ring of the indole moiety as the presence of another substituent appears to be not critical for *σ*_2_ affinity [30,31,32].

In light of the neurotoxic and tremorigenic effects, which associated with a complex structure prevent ibogaine from potential clinical applications, synthetic *σ*_2_ receptor analogs with low toxicity may be particularly beneficial. However, the development of ligands endowed with high affinity and selectivity can often run into several limitations and challenges. With the aim to overcome this issue, we recently reported the *σ*_2_ receptor selective ligand database (S2RSLDB, http://www.researchdsf.unict.it/S2RSLDB, accessed on 23 January 2019), a manually curated collection of the whole set of selective *σ*_2_ receptor ligands published in the literature [33]. At the same time, we also developed a 2D-QSAR affinity filter [34], built-up with 548 compounds, and a 3D-QSAR model for the identification of potentially selective *σ*_2_ receptor ligands [35,36].

With the interest to find new and easily synthesizable skeletons able to interact with the *σ*_2_ receptor, we have performed a deconstruction study of the ibogaine structure. In the study we report here, we have systematically modified the tricyclic moiety of ibogaine and its indole counterpart using a scaffold-hopping approach, and investigated the ability of the new obtained fragments to bind to the *σ*_2_ receptor. The *σ*_2_ p*K*_i_ values of these new compounds were predicted applying the abovementioned 3D-QSAR model and the potency of the best-scored molecules were further validated by a molecular docking analysis using the *σ*_2_ receptor homology model already reported by us [37].

## 2. Results and Discussion

### 2.1. 3D-Ligand Evaluation and Scaffold-Hopping Analysis

With the aim to produce a library of virtual compounds to further guide us in development of new hit ibogaine derived *σ*_2_ receptor ligands, we proceed to deconstruct the tricyclic ibogaine system containing the azepane moiety by a first scaffold-hopping [38,39,40] approach set out to maintain an indolo fused six- or seven-membered ring (Figure 1, Series 1). Successively, on the best-scored compound was performed a second scaffold-hopping cycle to alter the external aromatic (Figure 1, Series 2). As expected, this second series of compounds results as potentially more effective; in fact, in this series the ibogaine scaffold was optimized in both the selected components in Figure 1, differently in the series only one component was optimized.

Then the resulted molecules (1055 from Series 1 and 500 from Series 2) were filtered through statistical/2D descriptors filters using DataWarrior software [41]. To perform this, we analyzed the most potent and selective compounds present in the S2RSLDB [33] retrieving only the ligands presenting a *σ*_2_ *K*_i_ value ≤ 10 nM and a *σ*_1_/*σ*_2_ selectivity ≥ 1, for a total of 115 entities. The ranges of molecular weight (up to 651), cLogP (1.76/8.43), cLogS (−9.51/−2.26), H-acceptors (1/9), H-donor (0/2), Druglikeness (−15.1/8.2), DrugScore (0.04/0.86), topological polar surface area (3/96) belonging to the 115 potent and selective compounds were all chosen as 2D descriptors and the dataset of 1555 molecules was further filtered using these interval values to give 179 molecules from the Series 1 and 319 molecules from the Series 2.

The resulting 498 filtered molecules and the ibogaine were aligned in the 3D-QSAR model using Forge (v10.4.2, Cresset, New Cambridge House, United Kingdom) as a software [42], by adopting parameters reported in Figures S1 and S2. Once aligned, these compounds were scored assuming that if the fields (defined as the local extrema of the electrostatic, van der Waals, and hydrophobic potentials of each molecule) of the newly designed molecules are very similar to that of the original compounds, the resulting compounds will have similar biological properties [37,43,44,45]. The evaluation of the ibogaine in the 3D-QSAR model resulted in a predicted p*K*_i_ value of 6.8, which is in excellent agreement with the experimental one (6.69) [22]. Some selected compounds resulted from the 3D-ligand based filter are reported in Table 1; Table 2 while the full set of compounds is present in the Supplementary Materials (Tables S1 and S2). Overall, the results indicate that the double scaffold-hopping approach and the following 3D-QSAR model evaluation generate compounds with a suitable chemical structure for the *σ*_2_ receptor binding. Most importantly, several of the new generated compounds are predicted to be more effective than the parent hit compound ibogaine.

**Figure 2:**
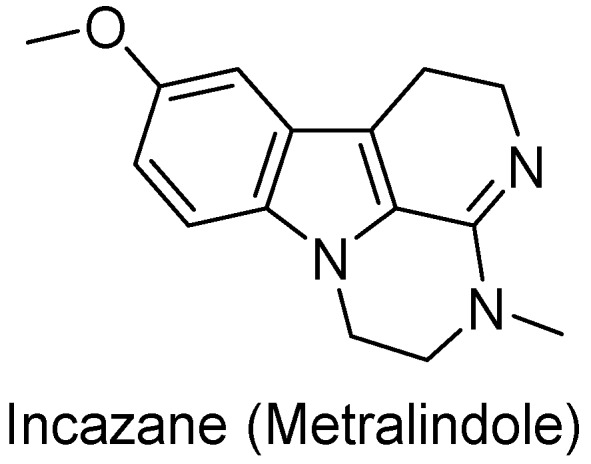
Structure of incazane.

Interestingly, among the 179 molecules of Series 1 we found a simplified analogue (Table 1) of incazane (metralindole, Figure 2), a reversible inhibitor of the monoamine oxidase A possessing an antidepressant activity [46], and the natural product pinoline (Table 1), another inhibitor of the monoamine oxidase A [47].

### 2.2. Molecular Docking Analysis

To further validate the predicted p*K*_i_ values of the 3D-QSAR model and to investigate the interactions of the new ligands within the *σ*_2_ receptor active site, we conducted a docking study on the selected compounds reported in Table 3. Each ligand was docked in the binding pocket of the *σ*_2_ receptor structure already built, in our group, by homology modeling [37]; successively, the best pose/receptor complex structure was minimized to allow the ligand to better adapt to the pocket of the active site and then a re-docking was performed using the same procedure already reported by us [48]. The values of the calculated p*K*_i_, reported in Table 3, are well in accord to the predicted ones by the 3D-QSAR model with the exception for the incazane derivative and compound 2_1. Interestingly, the p*K*_i_ value calculated by docking for compound 2_1 is the same as DTG (1,3-di(2-tolyl)guanidine) [49], a selective sigma receptor ligand used for the binding assays, of which it shares the portion similar to guanidine.

Moreover, to investigate the *σ*_1_/*σ*_2_ selectivity (SI) of this set of compounds, we conducted a molecular docking study using the crystal structure of the human *σ*_1_ receptor model bound to the high-affinity and selective *σ*_1_ antagonist PD144418 (PDB ID: 5HK1), employing the same methodology already validated by us [48]. The SI values reported in Table 3 for reference compounds (ibogaine and DTG) indicate that the computational models are efficient in the prediction and the new compounds should possess an effective *σ*_2_ selectivity.

**Figure 3:**
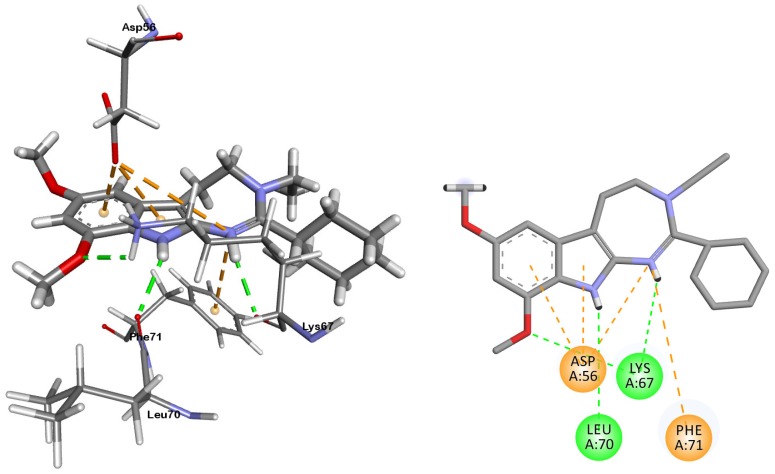
3D (left) and 2D (right) representations of the docked pose for compound 2_4. Green dotted lines represent hydrogen bonds and orange dotted lines *π-*ion interactions.

A representation of the best docked pose for compound 2_4 is depicted in Figure 3. There are clearly visible two hydrogen bonds between the LYS67 and LEU70 with the two hydrogen atoms at nitrogens and another one between LYS67 and a methoxyl oxygen atom. Moreover, two *π-*ion interactions were established between the ASP56 and the two aromatic rings of the indole and another two between the ASP56 and PHE71 with the *π-*orbital of the nitrogen atom of the 1,3-diazepine ring. A comparison of the best docked poses for ibogaine, pinoline and compound 2_4 are reported in Figure S3.

### 2.3. Pinoline Biological Assay

Among the compounds with the best 3D-QSAR predicted and docking calculated p*K*_i_ values, we decided to evaluate pinoline (compound 1_179) for affinity at both *σ*_1_ and *σ*_2_ receptors, with haloperidol as reference standard. Our choice was grounded on a structural simplicity, ease of commercial availability, and based on the fact that literature data for sigma binding affinity for pinoline have not been provided yet. However, the lack of a substituent on the *N*-atom of the piperidine appears to be critical for *σ*_2_ affinity since a *K*_i_ of 35.4 ± 2.6 µM (p*K*_i_ = 4.45) has been shown (Figure S4), thus confirming the range of magnitude for this displacement assay predicted by the in silico models. Moreover, the measure of the *σ*_1_ affinity for pinoline give a *K*_i_ value > 100 (p*K*_i_ < 4.00) accordingly with the calculated selectivity.

## 3. Materials and Methods

### 3.1. 2D to 3D Building and Minimization of Structures

The structures of ibogaine and related compounds were built using Marvin Sketch (ChemAxon, Budapest, Hungary). The 2D structures were subjected to molecular mechanics energy minimization by Merck molecular force field (MMFF94) using the Marvin Sketch geometrical descriptors plugin. The protonation states of the molecules were calculated considering a neutral pH. Before the alignment for the 3D-QSAR filter, the geometry of the obtained 3D structures was further optimized at semi-empirical level using the parameterized model number 3 (PM3) Hamiltonian [50,51] as implemented in MOPAC package (vMOPAC2016, Stewart Computational Chemistry, Colorado Springs, CO, USA) [52].

### 3.2. Compound Alignment and Scaffold-Hopping Analysis

All the optimized three-dimensional structures were imported into the software Forge (v10.4.2, Cresset, New Cambridge House, UK). The computational evaluation of all the molecules in the imported dataset was made by the field-based 3D-QSAR model previously published [35], after a careful alignment with the training set of the model (see Supplementary Material for more information). The molecules were described by means of field points (negative, positive, shape and hydrophobic), and all of them were generated using the extended electron distribution (XED) force field in Forge. In Figures S1 and S2 (Supplementary Material) are shown the software’s parameters used for the conformation hunt and the alignment. 500 was set as maximum number of conformations generated for each molecule. The root-mean-square deviation of atomic positions cutoff for duplicate conformers was set to 0.5 Å (the similarity threshold below which two conformers are assumed identical). The gradient cutoff for conformer minimization was set to 0.1 kcal/mol. The energy window was set to 2.5 kcal/mol. Conformers with a minimized energy outside the energy window were discarded. The scaffold-hopping analysis was performed using Spark as a software (v10.4.0) using the same 511717 fragments [38,39,40,45].

### 3.3. Molecular Docking

Docking experiments were performed employing AutoDock 4.2.5.2 software implemented in YASARA (v. 18.12.7, YASARA Biosciences GmbH, Vienna, Austria) [53,54] using the homology model of the *σ*_2_ receptor previously built by the same authors. The maps were generated by the program AutoGrid (4.2.5.2) with a spacing of 0.375 Å and dimensions that encompass all the surface of the active site. All the parameters were inserted at their default settings as previously reported [37]. 

To allow each ligand to adapt to the binding pocket, we carried out this study utilizing a three-step sequence already validated by us [48]: (i) ligand was docked upon *σ*_1_ or *σ*_2_ receptor, (ii) 5 ns of molecular dynamic (MD) simulation of the best pose obtained for the ligand/*σ* receptor complex, in order to accommodate the ligand, and (iii) redocking of the complex obtained from the last 3 ns of MD simulation averaged frames. The MD simulation was performed as described in Reference [48].

### 3.4. Radioligand Binding Assay

Sigma-2 binding experiments were performed as previously reported by Matsumoto et al. [55] and Mach et al. [56]. Briefly, each tube containing 360 µg of membrane protein was incubated with 3.26 nM [^3^H]DTG (1,3-di-2-tolylguanidine, Perkin Elmer, Waltham, MA, USA) (31 Ci/mM) in the presence of 400 nM (+)-SKF10,047 (Sigma-Aldrich, Saint Louis, MO, USA) to mask the *σ*_1_ sites. Test compounds were dissolved in dimethyl sulfoxide and then diluted in buffer to a final volume of 1 mL. Pinoline (Sigma-Aldrich, Saint Louis, MO, USA) was added to give a concentration in the range of 10^−3^–10^−10^ M, while haloperidol (Sigma-Aldrich, Saint Louis, MO, USA) was added to give a concentration in the range of 10^−5^–10^−10^ M. Incubation was carried out in 50 mM Tris-HCl (pH 8.0) for 120 min at room temperature. Each assay was terminated by the addition of ice-cold 10 mM Tris-HCl, pH 8.0, followed by filtration through a Whatman GF/B glass fiber filter that had been presoaked for 1 h in a 0.5% polyethylenimine (PEI) (Sigma-Aldrich, Saint Louis, MO, USA) solution. Filters were washed twice with 4 mL of ice-cold buffer. Non-specific binding was assessed in the presence of 5 µM DTG (Tocris, Minneapolis, MN, USA).

Sigma-1 binding assays were carried out according to DeHaven et al. [57]. Each tube containing 500 µg of membrane protein was incubated with 3.26 nM [^3^H]-(+)-pentazocine (Perkin Elmer, Waltham, MA, USA) (45 Ci/mmol) in 50 mM Tris-HCl (pH 7.4). Non-specific binding was evaluated in the presence of 10 µM haloperidol. Test compounds were dissolved in dimethyl sulfoxide and then diluted in buffer to a final volume of 1 mL. Pinoline was added to give a concentration of 10^−4^ M, while haloperidol was added to give a concentration in the range of 10^−5^–10^−10^ M. After incubation (150 min at 37 °C), the samples were filtered through Whatman GF/B glass fiber filters that were presoaked in a 0.5% PEI solution using a millipore filter apparatus. The filters were washed twice with 4 mL of ice-cold buffer and the amount of bound radioactivity on the filters air-dried and then soaked in Scintillation cocktail (Ultima Gold MV, Perkin Elmer, Waltham, MA, USA) was measured using a liquid scintillation counter (Beckman LS6500). Results are expressed as inhibition constants (*K*_i_ values) and calculated using GraphPad Prism (GraphPad Software, San Diego, CA, USA).

## 4. Conclusions

Ibogaine simplified analogs with high affinity for *σ*_2_ receptor represent an attractive and useful field to investigate. However, the development of ligands endowed with high affinity and selectivity has often several challenges. In this view, in silico methods have become essential tools in the drug design process. With the aim to find new, easily synthesizable skeletons able to interact with *σ*_2_ receptor, we here reported a deconstruction study on the ibogaine tricyclic moiety and a successive scaffold-hopping of the indole counterpart that indicated two new scaffolds that further decorated could constitute an excellent alternative for the synthesis of powerful *σ*_2_ receptor ligands. In particular, compound 2_4 emerged for the predicted/calculated p*K*_i_ values of 8.1 and 8.39, respectively, which are about 1.6 units higher than that of ibogaine. We eventually evaluated pinoline, a carboline derivative, for *σ*_2_ receptor affinity through radioligand binding assay and the result confirmed the predicted high µM range of affinity and even a good selectivity. The obtained results will be used by our research group for the next step in the development of new ibogaine simplified analogs with improved *σ*_2_ receptor binding capabilities.

## Figures and Tables

**Table 1:** Structure and predicted p*K*_i_ values of the selected ibogaine derivatives resulted from the scaffold-hopping study of Series 1.

Entry ID	Structure	Predicted p*K*_i_
1	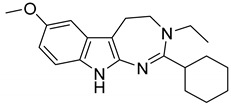	7.4
6	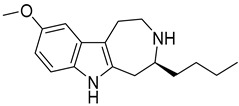	7.0
45	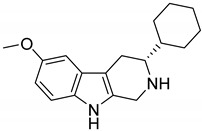	6.9
125 (Incazane derivative)	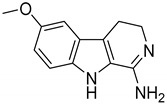	6.5
179 (Pinoline)	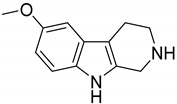	4.7

**Table 2:** Structure and predicted p*K*_i_ values of the selected ibogaine derivatives resulted from the scaffold-hopping study of Series 2.

Entry	Structure	Predicted p*K*_i_
1	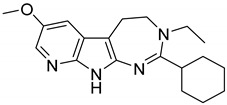	8.3
4	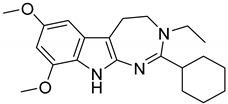	8.1
35	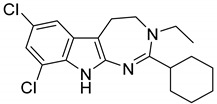	7.8

**Table 3:** Docking calculated *σ*_2_ p*K*_i_ values compared to the 3D-QSAR predicted ones and docking calculated *σ*_1_ p*K*_i_ values with *σ*_1_/*σ*_2_ selectivity index for selected compounds.

Series ID_Entry ID	3D-QSAR Predicted *σ*_2_ p*K*_i_	Docking Calculated *σ*_2_ p*K*_i_	Docking Calculated *σ*_1_ p*K*_i_	SI ^a^
1_1	7.4	7.24	6.50	5.5
1_6	7.0	6.98	6.81	1.5
1_45	6.9	7.19	6.77	2.6
1_125 (Incazane derivative)	6.5	7.40	5.13	186.2
1_179 (Pinoline)	4.7	4.53	3.81	5.2
2_1	8.3	7.56	6.15	25.7
2_4	8.1	8.39	6.65	55.0
2_35	7.8	8.14	6.58	36.3
Incazane	6.4	6.63	5.35	19.1
Ibogaine	6.8	6.89	5.06	67.6 ^b^
DTG	6.8	7.27	7.32	0.9 ^c^

^a^ SI: Selectivity index calculated as *σ*_1_ *K*_i_/*σ*_2_ *K*_i_. ^b^ SI = 42.5 from Reference [22]. ^c^ SI = 1.1 from Reference [49].

## Supplementary Materials

Supplementary materials can be found at http://www.mdpi.com/1422-0067/20/3/488/s1.
