# Supplementary material for: Morphing of Ibogaine: A Successful Attempt into the Search for Sigma-2 Receptor Ligands
Source: Int J Mol Sci. 2019 Jan 23;20(3):488. doi: 10.3390/ijms20030488 (PMC6386901; doi:10.3390/ijms20030488)
Supplement: Supplementary file 1 [file ijms-20-00488-s001.pdf]

International Journal of *Molecular Sciences*

Supplementary Materials

Table of content

Fig. S1. Forge's parameters used for the conformation hunt
Fig. S2. Forge's parameters used for the alignment
Table S1. Smiles and predicted pK_i values of the ibogaine derivatives resulted from the scaffold-hopping study of Series 1
Table S2. Smiles and predicted pK_i values of the ibogaine derivatives resulted from the scaffold-hopping study of Series 2
Figure S3. Comparison of the best docked poses for ibogaine, pinoline and compound 2_4
Figure S4. Binding curves. Haloperidol on σ_1 K_i = 3.2 nM (up-left) and σ_2 K_i = 102.2 nM (up-right) receptor. Pinoline on σ_2 receptor K_i = 35.4 mM (down); K_i for pinoline at σ_1 receptor >100 mM.

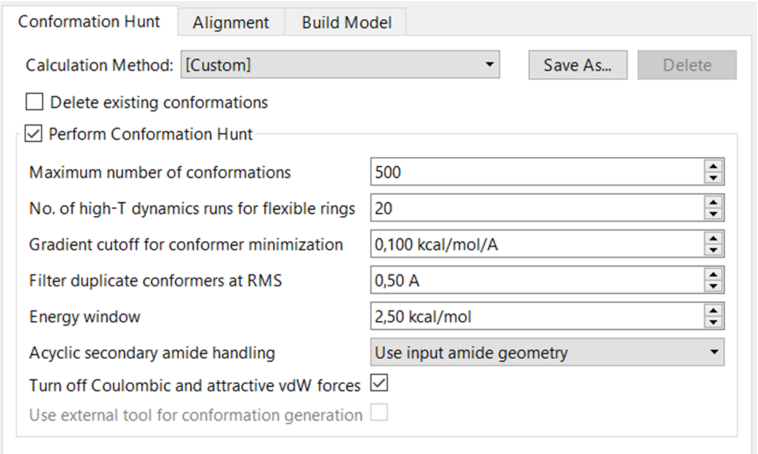

Figure S1. Forge's parameters used for the conformation hunt.

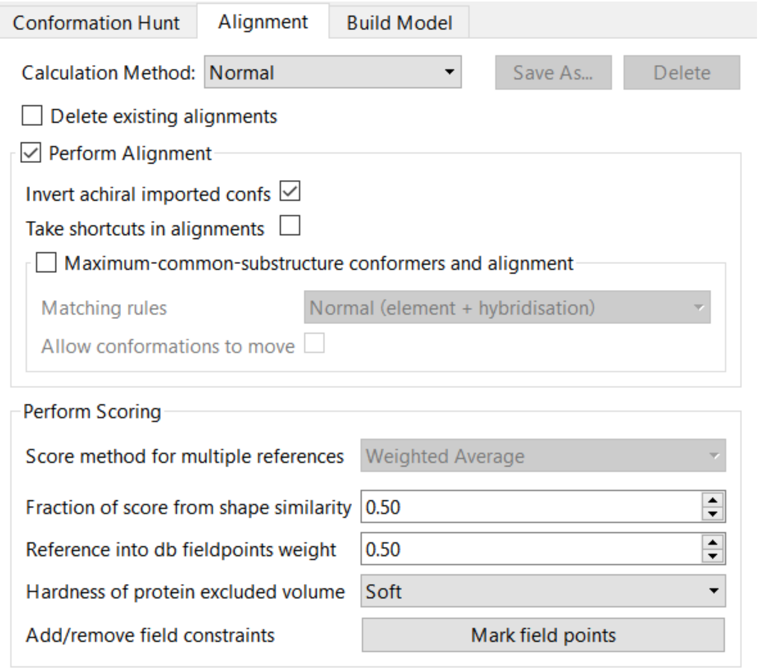

Figure S2. Forge's parameters used for the alignment.

Table S1. Smiles and predicted pK_i values of the ibogaine derivatives resulted from the scaffold-hopping study of Series 1.

ID	SMILES	pK_i
1	O(C)c1cc2c3CCN(CC)C(=[NH+]c3[nH]c2cc1)C1CCCCC1	7.4
2	O(C)c1cc2c3CCN(CC)C(=[NH+]c3[nH]c2cc1)C(C)C	7.3
3	O(C)c1cc2c3CC[N@H+]([C@H]4[C@H](CCCCC4)c3[nH]c2cc1)CC	7.1
4	O(C)c1cc2c3[C@@H]4[C@H](CC=CC4)C[NH2+]Cc3[nH]c2cc1	7.1
5	O(C)c1cc2c3CC[C@@H](C)[C@@H]([NH2+]CCC)Cc3[nH]c2cc1	7.0
6	O(C)c1cc2c3CC[NH2+][C@H](Cc3[nH]c2cc1)CCCC	7.0
7	O(C)c1cc2c3CCC[C@@H]([N@@H+]4CCCC[C@H]4C)Cc3[nH]c2cc1	7.0
8	s1c(ccc1C)[C@@H]1[N@@H+](CCc2c([nH]c3c2cc(OC)cc3)C1)C	7.0
9	O(C)c1cc2c3C[C@@]4([NH2+]Cc3[nH]c2cc1)CCCC=C4	7.0
10	Br[C@@H]1c2c([nH]c3c2cc(OC)cc3)C[NH2+][C@H]1Br	7.0
11	O(C)c1cc2c3CC[C@@H]4[C@@H](C[C@@H]([NH+](C)C)C4)c3[nH]c2cc1	7.0
12	O(C)c1cc2c3C[C@@H]([NH2+]Cc3[nH]c2cc1)C(CC)CC	7.0
13	FC(F)(F)C[C@@H]1[NH2+]Cc2[nH]c3c(cc(OC)cc3)c2C1	7.0
14	O(C)c1cc2c3CC[NH2+][C@H]4[C@H](CCCC4(C)C)c3[nH]c2cc1	7.0
15	O(C)c1cc2c3C[C@@H]([NH2+]Cc3[nH]c2cc1)CCC(C)C	7.0
16	O(C)c1cc2c3CCC[C@@H]([NH+]4CCC(CC4)C)Cc3[nH]c2cc1	7.0
17	O(C)c1cc2c3C[C@@H]([NH2+]Cc3[nH]c2cc1)CCCCC	7.0
18	O(C)c1cc2c3C[C@@H]([NH2+]Cc3[nH]c2cc1)CC(C)(C)C	7.0
19	O(C)c1cc2c3C[C@@H]([NH2+]Cc3[nH]c2cc1)C1CCCC1	7.0
20	O(C)c1cc2c3CC[N@H+]4CC[C@@H](c3[nH]c2cc1)[C@@H]4Cc1ccccc1	7.0
21	O(C)c1cc2c3C[C@@H]([NH2+]Cc3[nH]c2cc1)[C@H](CCC)C	7.0
22	o1cccc1C[C@@H]1[NH2+]Cc2[nH]c3c(cc(OC)cc3)c2C1	7.0
23	S1[C@H]2[C@@H]([NH2+]Cc3[nH]c4c(cc(OC)cc4)c23)CC1	7.0
24	S1[NH2+]Cc2[nH]c3c(cc(OC)cc3)c2-c2occc12	7.0
25	O(C)c1cc2c3[C@@H]4[C@@H]([NH2+]Cc3[nH]c2cc1)CCCCC4	7.0
26	O(C)c1cc2c3[C@@H]4[C@@H](CCCC4)C[NH2+]Cc3[nH]c2cc1	7.0
27	Br[C@@H]1[NH2+]Cc2[nH]c3c(cc(OC)cc3)c2C1	6.9
28	O(C)c1cc2c3CC[N@H+]4CCCC[C@@H](c3[nH]c2cc1)[C@@H]4C(C)C	6.9
29	O(C)c1cc2c3CC[N@@H+]4[C@H](Cc3[nH]c2cc1)c1c(CC4)cccc1	6.9
30	Clc1cc(ccc1)[C@@H]1[N@@H+](CCc2c([nH]c3c2cc(OC)cc3)C1)C	6.9
31	S(CC)[C@@H]1[NH2+]Cc2[nH]c3c(cc(OC)cc3)c2C1	6.9
32	S1[C@@H]2CCc3c4cc(OC)ccc4[nH]c3[C@H](C1)[C@@H]2O	6.9
33	S1CCC[C@]12[NH2+]Cc1[nH]c3c(cc(OC)cc3)c1C2	6.9
34	O(C)c1cc2c3CC[C@@H](O)CC(Cc3[nH]c2cc1)(C)C	6.9
35	o1cccc1[C@@H]1[NH2+]CCc2c([nH]c3c2cc(OC)cc3)C1	6.9
36	O(C)c1cc2c3C[C@]4([NH2+]Cc3[nH]c2cc1)C[C@H](CC4)C	6.9
37	O(C)c1cc2c3CC[C@@H]4[C@@H](CCC[NH2+]C4)Cc3[nH]c2cc1	6.9
38	S(C[C@@H]1[NH2+]Cc2[nH]c3c(cc(OC)cc3)c2C1)CC	6.9
39	ClC(Cl)(Cl)[C@@H]1[NH2+]Cc2[nH]c3c(cc(OC)cc3)c2C1	6.9
40	O(C)c1cc2c3C[C@@]4([NH2+]Cc3[nH]c2cc1)CCC=CC4	6.9
41	FC(F)(F)[C@H]1[NH2+]Cc2[nH]c3c(cc(OC)cc3)c2[C@@H]1CC	6.9
42	O(C)c1cc2c3CC4([NH2+]Cc3[nH]c2cc1)CCCCCC4	6.9
43	Clc1cc(ccc1)[C@@H]1[N@H+]2CCc3c4cc(OC)ccc4[nH]c3N1CC2	6.9
44	O(C)c1cc2c3CC[N@@H+]4[C@@H](C=5C6CCC(C=5CC4)CC6)Cc3[nH]c2cc1	6.9
45	O(C)c1cc2c3C[C@@H]([NH2+]Cc3[nH]c2cc1)C1CCCCC1	6.9
46	Clc1ccc(cc1)[C@@H]1[N@H+]2CCc3c4cc(OC)ccc4[nH]c3N1CC2	6.9

47	Fc1cc2-c3[nH]c4c(cc(OC)cc4)c3CCNc2cc1C	6.9
48	O(C)c1cc2c3CC[NH+]=C(N(c3[nH]c2cc1)C)C1CCCCC1	6.9
49	Clc1cc(Cl)cc2c1[C@@H]1[N@@H+](CCc3c([nH]c4c3cc(OC)cc4)C1)C2	6.9
50	S1[C@H]2[C@H](SC1(C)C)[NH2+]Cc1[nH]c3c(cc(OC)cc3)c12	6.9
51	O(C)c1cc2c3[C@@H]4[C@H]([NH2+]Cc3[nH]c2cc1)CCC=C4C	6.9
52	O(C)c1cc2c3CC[N@H+]4CC[C@@H](c3[nH]c2cc1)[C@@H]4C1CCCC1	6.8
53	s1cccc1[C@@H]1[NH2+]CCc2c([nH]c3c2cc(OC)cc3)C1	6.8
54	S1[C@@H]2[C@@H](CCOC2)c2[nH]c3c(cc(OC)cc3)c2CC1	6.8
55	S1CCSC12[NH2+]Cc1[nH]c3c(cc(OC)cc3)c1C2	6.8
56	O(C)c1cc2c3CC4([NH2+]Cc3[nH]c2cc1)CC1(C4)CC1	6.8
57	FC(F)(F)[C@@H]1c2c([nH]c3c2cc(OC)cc3)C[NH2+][C@@H]1C	6.8
58	SCCC[C@@H]1[NH2+]Cc2[nH]c3c(cc(OC)cc3)c2C1	6.8
59	S(CC[C@@H]1[NH2+]Cc2[nH]c3c(cc(OC)cc3)c2C1)C	6.8
60	O(C)c1cc2c3C[C@]4([NH2+]Cc3[nH]c2cc1)CCC[C@H]4C	6.8
61	O(C)c1cc2c3C[C@@]([NH2+]Cc3[nH]c2cc1)(CC(C)C)C	6.8
62	O(C)c1cc2c3C[C@]4([NH2+]Cc3[nH]c2cc1)C[C@@H](CC4)CC	6.8
63	O(C)c1cc2c3CC[C@H](O)[C@@H]4[C@@H](CC=CC4)c3[nH]c2cc1	6.8
64	O(C)c1cc2c3CC[N@@H+]4CCC[C@H](c3[nH]c2cc1)[C@H]4C(C)C	6.8
65	O(C)c1cc2c3CCC[C@@H]([NH+]4CCCCCC4)Cc3[nH]c2cc1	6.8
66	O(C)c1cc2c3CC[NH2+][C@@H](Cc3[nH]c2cc1)C(C)(C)C	6.8
67	O(C)c1cc2c3CCC[C@@H]([NH2+]CCC)Cc3[nH]c2cc1	6.8
68	S1[C@H]2[C@H]([NH2+]CC1)CCc1c([nH]c3c1cc(OC)cc3)C2	6.8
69	O(C)c1cc2c3CC[N@@H+](C)[C@H](Cc3[nH]c2cc1)c1ccc(cc1)C	6.8
70	S1[C@@H]2[NH2+]Cc3[nH]c4c(cc(OC)cc4)c3[C@H]2CCC1	6.8
71	O1[C@H]2[C@H](c3[nH]c4c(cc(OC)cc4)c3CC2)C(=C)[C@H]1CO	6.8
72	O(C)c1cc2c3CC[NH2+][C@H](Cc3[nH]c2cc1)CC(C)C	6.8
73	o1c-2c(cc1C)[C@H]([NH2+]CC)CCc1c-2[nH]c2c1cc(OC)cc2	6.8
74	S1[NH2+]Cc2[nH]c3c(cc(OC)cc3)c2[C@@H]2[C@@H]1CCC2	6.8
75	O(C)c1cc2c3CC[N@H+]4CCCC[C@@H](c3[nH]c2cc1)[C@@H]4CC	6.7
76	O(C)c1cc2c3CC[NH2+][C@@](Cc3[nH]c2cc1)(C(C)C)C	6.7
77	S(C)C1=[NH+]CCc2c3cc(OC)ccc3[nH]c2N1C	6.7
78	SC[C@H]1c2c([nH]c3c2cc(OC)cc3)C[NH2+][C@H]1C	6.7
79	FC(F)(F)[C@@]1([NH2+]Cc2[nH]c3c(cc(OC)cc3)c2C1)C	6.7
80	O(C)c1cc2c3CC[NH2+][C@H](Cc3[nH]c2cc1)c1ccccc1	6.7
81	O(C)c1cc2c3CCNc4c(-c3[nH]c2cc1)cccc4	6.7
82	Br[C@H](CC1=[NH+]CCc2c3cc(OC)ccc3[nH]c2N1C)C	6.7
83	O(C)c1cc2c3c([nH]c2cc1)C[NH2+][C@@H](C)C13CCCC1	6.7
84	Fc1c2NCCc3c([nH]c4c3cc(OC)cc4)-c2ccc1	6.7
85	o1c-2c(cc1)[C@H]([NH2+]CC)CCc1c-2[nH]c2c1cc(OC)cc2	6.7
86	Fc1ccc(cc1)[C@@H]1[NH2+]CCc2c([nH]c3c2cc(OC)cc3)C1	6.7
87	Fc1cc2-c3[nH]c4c(cc(OC)cc4)c3CCNc2cc1	6.7
88	O(C)c1cc2c3c([nH]c2cc1)C[NH2+][C@H](C)[C@@H]3C(C)(C)C	6.7
89	s1cc(cc1)[C@H]1[NH2+]Cc2[nH]c3c(cc(OC)cc3)c2C1	6.7
90	O(C)c1cc2c3c([nH]c2cc1)C1(CCC3)CC[NH2+]CC1	6.7
91	O(C)c1cc2c3CC[N@H+]4[C@H]5[C@H](CCCC5)C[C@@H](c3[nH]c2cc1)C4	6.7
92	O1CCCC([NH2+]CCc2c3cc(OC)ccc3[nH]c12)(C)C	6.7
93	Fc1cc2-c3[nH]c4c(cc(OC)cc4)c3CCNc2nc1	6.7
94	Clc1cc2-c3[nH]c4c(cc(OC)cc4)c3CCNc2nc1	6.7
95	O(C)c1cc2c3[C@H]4[C@@H]5CC[C@H]([C@H]4[NH2+]Cc3[nH]c2cc1)C5	6.7

96	Fc1ccc(cc1)[C@@H]1[N@H+]2CCc3c4cc(OC)ccc4[nH]c3[C@@H]1CC2	6.6
97	S1[C@H]2c3[nH]c4c(cc(OC)cc4)c3CC[N@@H+](CC1)[C@H]2c1ccccc1	6.6
98	O1CC[C@@H]2[NH2+]CCc3c4cc(OC)ccc4[nH]c3[C@@H]2C1	6.6
99	O(C)c1cc2c3CC[NH2+][C@@H]4[C@@H](CCCc5c4cccc5)c3[nH]c2cc1	6.6
100	Cl\C(\Cl)=C\[C@@H]1[C@H]2CCc3c([nH]c4c3cc(OC)cc4)[C@@]12C	6.6
101	O(C)c1cc2c3CCC[C@@H](Cc3[nH]c2cc1)C(C)(C)C	6.6
102	O(C)c1cc2c3C[C@@H](OCCC)[NH2+]Cc3[nH]c2cc1	6.6
103	Clc1cc2NCCc3c([nH]c4c3cc(OC)cc4)-c2cc1	6.6
104	O(C)c1cc2c3c([nH]c2cc1)C[NH2+][C@H](C)[C@@H]3[C@@H](CC)C	6.6
105	O(C)c1cc2c3c([nH]c2cc1)C[NH2+][C@H](C)[C@@H]3CC(C)C	6.6
106	O(C)c1cc2c3c([nH]c2cc1)C[NH2+]CC13CCCCC1	6.6
107	O(C)c1cc2c3c([nH]c2cc1)C[NH2+]C[C@@]3(CC)C1CC1	6.6
108	O(C)c1cc2c3CC[C@H](CC(Cc3[nH]c2cc1)(C)C)C	6.6
109	O(C)c1cc2c3CCC[C@@]4(Cc3[nH]c2cc1)CC[NH2+]C4	6.6
110	Clc1cc2-c3[nH]c4c(cc(OC)cc4)c3CCNc2cc1	6.6
111	O(C)c1cc2c3CC[NH+]=C(N(c3[nH]c2cc1)C)CCC	6.6
112	Fc1ccc(cc1)C1=[NH+]CCc2c3cc(OC)ccc3[nH]c2N1C	6.6
113	S1[C@H]2[C@@H](SCC1)[NH2+]Cc1[nH]c3c(cc(OC)cc3)c12	6.6
114	O(C)c1cc2c3CCC[C@@H]4N(CC[NH2+]C4)c3[nH]c2cc1	6.6
115	S1C2=[NH+]CCc3c4cc(OC)ccc4[nH]c3N2C[C@H]1C	6.6
116	O(C)c1cc2c3CCNc4cc(ccc4-c3[nH]c2cc1)C	6.6
117	O(C)c1cc2c3N4[C@H]([NH2+]Cc3[nH]c2cc1)CCC[C@H]4C	6.6
118	Fc1cc2c(C[C@@H]3[N@@H+]4CC[C@]2([C@H]3c2[nH]c3c(cc(OC)cc3)c2CC4)C)cc1	6.6
119	Brc1cc2NCCc3c([nH]c4c3cc(OC)cc4)-c2cc1	6.6
120	O(C)c1cc2c3[C@@H]4[C@@H]5[C@H]([NH2+]Cc3[nH]c2cc1)C[C@H](C4)C5	6.6
121	O(C)c1cc2c3[C@H]4[C@H]5C=C[C@@H]([C@H]4[NH2+]Cc3[nH]c2cc1)C5	6.6
122	O(C)c1cc2c3CC[N@H+]4CCCC[C@@H](c3[nH]c2cc1)[C@@H]4C1CC1	6.5
123	S1[C@@H]2CCc3c4cc(OC)ccc4[nH]c3[C@H](C1)[C@@H]2F	6.5
124	O(C)c1cc2c3CC[C@H]4[NH2+]CCN(c3[nH]c2cc1)[C@@H]4C	6.5
125	NC1=[NH+]CCC2=C1N(C3=C2C=C(OC)C=C3)[H]	6.5
126	s1c2NCCc3c([nH]c4c3cc(OC)cc4)-c2cc1	6.5
127	O(C)c1cc2c3CC[NH2+]C[C@](Cc3[nH]c2cc1)(CCC)C	6.5
128	O(C)c1cc2c3CC[C@H]4CCC[C@]4(Cc3[nH]c2cc1)C	6.5
129	O1[C@@H]2[C@@H](CCC[C@@H]2O)c2[nH]c3c(cc(OC)cc3)c2CC1	6.5
130	Clc1cc2-c3[nH]c4c(cc(OC)cc4)c3CCNc2sc1	6.5
131	S1[C@H]2[NH2+][C@H](c3[nH]c4c(cc(OC)cc4)c3CC2)C1	6.5
132	Cl\C=C\[C@@H]1[C@H]2CCc3c([nH]c4c3cc(OC)cc4)[C@@]12C	6.5
133	S1CCCN2c3[nH]c4c(cc(OC)cc4)c3CC[NH+]=C12	6.5
134	Clc1ccc(cc1F)[C@@H]1[N@H+]2CCc3c4cc(OC)ccc4[nH]c3[C@@H]1CC2	6.5
135	S1CCN2c3[nH]c4c(cc(OC)cc4)c3CC[NH+]=C12	6.5
136	O(C)c1cc2c3CCNc4c(-c3[nH]c2cc1)ccnc4	6.5
137	O(C)c1cc2c3CCCC4(Cc3[nH]c2cc1)CC[NH2+]CC4	6.5
138	O(C)c1cc2c3CC[C@]4([C@]5([C@@H]4C[NH2+]C5)c3[nH]c2cc1)C	6.5
139	O(C)c1cc2c3c([nH]c2cc1)C[NH2+][C@H](C(C)C)C13CC1	6.5
140	s1cc2-c3[nH]c4c(cc(OC)cc4)c3CCNc2c1	6.5
141	O(C)c1cc2c3CC[NH2+][C@H]4[C@H](CCCC4)Cc3[nH]c2cc1	6.5
142	O(C)c1cc2c3CC[C@@H]4C[C@@H](CC[C@@H]4C)c3[nH]c2cc1	6.5
143	O(C)c1cc2c3CC[C@@H]4[C@@H](CCCCC4)c3[nH]c2cc1	6.5
144	O(C)c1cc2c3CC[C@H]([NH2+]C)c4c(cc(cc4)C)-c3[nH]c2cc1	6.5

145	o1c-2c(C[NH2+]CCc3c-2[nH]c2c3cc(OC)cc2)c2c1cccc2	6.5
146	Clc1c2NCCc3c([nH]c4c3cc(OC)cc4)-c2ccc1	6.5
147	O(C)c1cc2c3CC[NH2+]C[C@@H](Cc3[nH]c2cc1)C(C)C	6.5
148	O(C)c1cc2c3CCCc4c(-c3[nH]c2cc1)cncc4	6.5
149	O(C)c1cc2c3CCC[C@@]4(CCC[NH2+]C4)Cc3[nH]c2cc1	6.5
150	S=C1NCCc2c3cc(OC)ccc3[nH]c2N1CC	6.5
151	S1C[C@H](c2[nH]c3c(cc(OC)cc3)c2CC1)[C@@H](F)C	6.4
152	O(C)c1cc2c3CC[C@H]4[C@H]5CC[C@H]([C@@H]4CC5)c3[nH]c2cc1	6.3
153	O1CCc2c([nH]c3c2cc(OC)cc3)-c2ncc(cc12)C	6.3
154	s1c2-c3[nH]c4c(cc(OC)cc4)c3CCNc2nc1	6.3
155	O(C)c1cc2c3c([nH]c2cc1)[C@]1([C@H]([NH2+]CC1)CC3)CC	6.3
156	S1c2[nH]c3c(cc(OC)cc3)c2CCCC1(C)C	6.3
157	O(C)c1cc2c3c([nH]c2cc1)[C@H](C)[C@@H]([NH+](C)C)CCC3	6.3
158	O(C)c1cc2c3CCNc4[n+](-c3[nH]c2cc1)cccc4	6.3
159	O(C)c1cc2c3CCC[C@@H]4C(=CCCC4)c3[nH]c2cc1	6.3
160	FC(F)(F)[C@@]12[C@@H](C1)CCc1c2[nH]c2c1cc(OC)cc2	6.3
161	O(C)c1cc2c3CC[C@H]4C=C(C[C@@H](c3[nH]c2cc1)C4)C	6.3
162	S1CC(=S)CCc2c([nH]c3c2cc(OC)cc3)C1	6.3
163	O(C)c1cc2c3CCC[C@]45[C@H](c3[nH]c2cc1)C[C@H](CC4)C5(C)C	6.3
164	O(C)c1cc2c3[C@@H]4CCC[C@@]4(C[NH2+]Cc3[nH]c2cc1)C	6.3
165	O1[C@H]2[C@H](CCCC2)CCc2c3cc(OC)ccc3[nH]c12	6.2
166	S1c2[nH]c3c(cc(OC)cc3)c2CCCC12CCC2	6.2
167	S1(=O)(=O)CCc2c([nH]c3c2cc(OC)cc3)C[C@@H]1c1occc1	6.2
168	Br[C@]12C[C@]1(CCc1c2[nH]c2c1cc(OC)cc2)C	6.2
169	F[C@H]1[C@H]2[C@@H]([NH2+]CCc3c4cc(OC)ccc4[nH]c23)CC1	6.2
170	S1CSc2[nH]c3c(cc(OC)cc3)c2CCC1=S	6.2
171	S=C1CCc2c3cc(OC)ccc3[nH]c2S(=O)(=O)[C@@H]1CC	6.1
172	S1CCc2c3cc(OC)ccc3[nH]c2SSC1	6.1
173	s1c2CCCc3c([nH]c4c3cc(OC)cc4)-c2nc1O	6.1
174	S1C[C@@H](CCc2c3cc(OC)ccc3[nH]c12)CF	6.1
175	FC(F)(F)[C@@]1(O)CCCc2c([nH]c3c2cc(OC)cc3)C1	6.1
176	S1(=O)(=O)N(CCCc2c3cc(OC)ccc3[nH]c12)C(C)(C)C	6.1
177	Clc1cc2CCCc3c([nH]c4c3cc(OC)cc4)-c2nc1	6.0
178	S1(=O)(=O)c2[nH]c3c(cc(OC)cc3)c2CCCC12CC2	5.9
179	COC1=CC2=C(NC3=C2CC[NH2+]C3)C=C1	4.7

Table S2. Smiles and predicted pK_i values of the ibogaine derivatives resulted from the scaffold-hopping study of Series 2.

ID	SMILES	pK_i
1	O(C)c1cc2c3CCN(CC)C(=[NH+]c3[nH]c2nc1)C1CCCCC1	8.3
2	O(C)c1cc(c2[nH]c3[NH+]=C(N(CCc3c2c1)CC)C1CCCCC1)C#N	8.3
3	O(C(C)C)c1cc2c3CCN(CC)C(=[NH+]c3[nH]c2nc1)C1CCCCC1	8.3
4	O(C)c1cc(OC)cc2c1[nH]c1[NH+]=C(N(CCc12)CC)C1CCCCC1	8.1
5	s1cc2c([nH]c3[NH+]=C(N(CCc23)CC)C2CCCCC2)c1C	8.1
6	s1cc2c3CCN(CC)C(=[NH+]c3[nH]c2c1)C1CCCCC1	8.0
7	Clc1c2[nH]c3[NH+]=C(N(CCc3c2sc1)CC)C1CCCCC1	8.0
8	FC(F)(F)c1cc(OC)cc2c1[nH]c1[NH+]=C(N(CCc12)CC)C1CCCCC1	8.0
9	[NH+]=1c2[nH]c3c(c2CCN(CC)C=1C1CCCCC1)cccc3C=C	8.0
10	s1cc2[nH]c3[NH+]=C(N(CCc3c2c1SC)CC)C1CCCCC1	8.0

11	Fc1c2c([nH]c3[NH+]=C(N(CCc23)CC)C2CCCCC2)c(F)cc1	8.0
12	Fc1cc(F)c2[nH]c3[NH+]=C(N(CCc3c2c1F)CC)C1CCCCC1	8.0
13	Clc1cc(F)c2[nH]c3[NH+]=C(N(CCc3c2c1)CC)C1CCCCC1	7.9
14	Clc1cc(OC(F)F)cc2c1[nH]c1[NH+]=C(N(CCc12)CC)C1CCCCC1	7.9
15	Fc1cc(OC(F)F)cc2c1[nH]c1[NH+]=C(N(CCc12)CC)C1CCCCC1	7.9
16	Sc1cc(F)cc2c1[nH]c1[NH+]=C(N(CCc12)CC)C1CCCCC1	7.9
17	Clc1cc(F)c2[nH]c3[NH+]=C(N(CCc3c2c1F)CC)C1CCCCC1	7.9
18	s1cc(c2[nH]c3[NH+]=C(N(CCc3c12)CC)C1CCCCC1)C	7.9
19	Clc1cc2c([nH]c3[NH+]=C(N(CCc23)CC)C2CCCCC2)c(F)c1C	7.9
20	S(C)c1cc(cc2c1[nH]c1[NH+]=C(N(CCc12)CC)C1CCCCC1)CC	7.9
21	s1c2c([nH]c3[NH+]=C(N(CCc23)CC)C2CCCCC2)c(C)c1C	7.9
22	Fc1cc(N(C)C)cc2c1[nH]c1[NH+]=C(N(CCc12)CC)C1CCCCC1	7.9
23	Brc1cc(F)cc2c1[nH]c1[NH+]=C(N(CCc12)CC)C1CCCCC1	7.9
24	O(C)c1c2c3CCN(CC)C(=[NH+]c3[nH]c2ncc1OC)C1CCCCC1	7.9
25	Fc1cc(c2[nH]c3[NH+]=C(N(CCc3c2c1)CC)C1CCCCC1)CC	7.9
26	[NH+]=1c2[nH]c3c(cc(cc3CC)C)c2CCN(CC)C=1C1CCCCC1	7.9
27	Brc1c2[nH]c3[NH+]=C(N(CCc3c2sc1)CC)C1CCCCC1	7.9
28	s1cc(c2[nH]c3[NH+]=C(N(CCc3c12)CC)C1CCCCC1)CC	7.9
29	FCOc1cc(c2[nH]c3[NH+]=C(N(CCc3c2c1)CC)C1CCCCC1)C	7.8
30	[NH+]=1c2[nH]c3c(cc(cc3C)C#C)c2CCN(CC)C=1C1CCCCC1	7.8
31	Fc1cc(OC(C)C)cc2c1[nH]c1[NH+]=C(N(CCc12)CC)C1CCCCC1	7.8
32	[NH+]=1c2[nH]c3c(cc(cc3C)C)c2CCN(CC)C=1C1CCCCC1	7.8
33	Clc1cc(OCC)cc2c1[nH]c1[NH+]=C(N(CCc12)CC)C1CCCCC1	7.8
34	[NH+]=1c2[nH]c3c(c2CCN(CC)C=1C1CCCCC1)cccc3C	7.8
35	Clc1cc(Cl)cc2c1[nH]c1[NH+]=C(N(CCc12)CC)C1CCCCC1	7.8
36	Clc1cc(c2[nH]c3[NH+]=C(N(CCc3c2c1C)CC)C1CCCCC1)C	7.8
37	[NH+]=1c2[nH]c3c(cc(cc3CC)CC)c2CCN(CC)C=1C1CCCCC1	7.8
38	Fc1ccc2c([nH]c3[NH+]=C(N(CCc23)CC)C2CCCCC2)c1C	7.8
39	O(C)c1cc2c3CCN(CC)C(=[NH+]c3[nH]c2nc1OC)C1CCCCC1	7.8
40	[NH+]=1c2[nH]c3c(c2CCN(CC)C=1C1CCCCC1)cccc3CC	7.8
41	Fc1c2c([nH]c3[NH+]=C(N(CCc23)CC)C2CCCCC2)c(cc1)C	7.8
42	[NH+]=1c2[nH]c3c(c2CCN(CC)C=1C1CCCCC1)ccc(C)c3C	7.8
43	[NH+]=1c2[nH]c3c(c2CCN(CC)C=1C1CCCCC1)c(ccc3)C#C	7.8
44	[NH+]=1c2[nH]c3c(c2CCN(CC)C=1C1CCCCC1)c(ccc3C)C	7.8
45	Brc1sc2c([nH]c3[NH+]=C(N(CCc23)CC)C2CCCCC2)c1C	7.8
46	Clc1c2[nH]c3[NH+]=C(N(CCc3c2cc(Cl)c1C)CC)C1CCCCC1	7.8
47	Fc1cc(c2[nH]c3[NH+]=C(N(CCc3c2c1F)CC)C1CCCCC1)C	7.8
48	[NH+]=1C=2Nn3c(cc(C)c3C)C=2CCN(CC)C=1C1CCCCC1	7.8
49	Fc1cc(OC)cc2c1[nH]c1[NH+]=C(N(CCc12)CC)C1CCCCC1	7.7
50	O(C)c1cc(c2[nH]c3[NH+]=C(N(CCc3c2c1)CC)C1CCCCC1)C	7.7
51	Clc1cc(OC)cc2c1[nH]c1[NH+]=C(N(CCc12)CC)C1CCCCC1	7.7
52	Sc1cc(c2[nH]c3[NH+]=C(N(CCc3c2c1)CC)C1CCCCC1)C	7.7
53	Fc1cc2[nH]c3[NH+]=C(N(CCc3c2cc1CC)CC)C1CCCCC1	7.7
54	[NH+]=1c2[nH]c3c(cc(cc3C)CC)c2CCN(CC)C=1C1CCCCC1	7.7
55	O(CC)c1cc(c2[nH]c3[NH+]=C(N(CCc3c2c1C)CC)C1CCCCC1)C	7.7
56	Clc1cc(c2[nH]c3[NH+]=C(N(CCc3c2c1)CC)C1CCCCC1)C	7.7
57	Fc1cc(OCC)cc2c1[nH]c1[NH+]=C(N(CCc12)CC)C1CCCCC1	7.7
58	O(CC)c1cc2c([nH]c3[NH+]=C(N(CCc23)CC)C2CCCCC2)c(C)c1C	7.7
59	O(C(C)C)c1cc(c2[nH]c3[NH+]=C(N(CCc3c2c1)CC)C1CCCCC1)C	7.7

60	Clc1cc(OCF)cc2c1[nH]c1[NH+]=C(N(CCc12)CC)C1CCCCC1	7.7
61	Clc1c2c([nH]c3[NH+]=C(N(CCc23)CC)C2CCCCC2)c(cc1OC)C	7.7
62	Clc1cc(OCCC)cc2c1[nH]c1[NH+]=C(N(CCc12)CC)C1CCCCC1	7.7
63	O(C(C)C)c1cc(c2[nH]c3[NH+]=C(N(CCc3c2c1C)CC)C1CCCCC1)C	7.7
64	O(C)c1cc(cc2c1[nH]c1[NH+]=C(N(CCc12)CC)C1CCCCC1)C=C	7.7
65	Fc1cc(cc2c1[nH]c1[NH+]=C(N(CCc12)CC)C1CCCCC1)[C@@H]1OCCC1	7.7
66	O(CC)c1c(C)c(c2[nH]c3[NH+]=C(N(CCc3c2c1C)CC)C1CCCCC1)C	7.7
67	o1c2c(c3[nH]c4[NH+]=C(N(CCc4c3cc2)CC)C2CCCCC2)cc1	7.7
68	Clc1cc2c([nH]c3[NH+]=C(N(CCc23)CC)C2CCCCC2)c(C)c1C	7.7
69	Fc1cc2[nH]c3[NH+]=C(N(CCc3c2cc1OCC)CC)C1CCCCC1	7.7
70	[NH+]=1C=2Nc3n(cc(C)c3C)C=2CCN(CC)C=1C1CCCCC1	7.7
71	O1CCc2c3[nH]c4[NH+]=C(N(CCc4c3ccc12)CC)C1CCCCC1	7.7
72	O(C)c1cc(c2[nH]c3[NH+]=C(N(CCc3c2c1)CC)C1CCCCC1)CCC	7.7
73	O1c2c3[nH]c4[NH+]=C(N(CCc4c3ccc2OC1)CC)C1CCCCC1	7.7
74	O(CC=C)c1cc(c2[nH]c3[NH+]=C(N(CCc3c2c1)CC)C1CCCCC1)C	7.7
75	O(C(C)C)c1cc2c([nH]c3[NH+]=C(N(CCc23)CC)C2CCCCC2)c(C)c1C	7.7
76	S1S[C@H]2N[C@H]3[NH+]=C(N(CC[C@H]3[C@H]2[C@H]1SC)CC)C1CCCCC1	7.7
77	[NH+]=1C=2Nn3c(C=2CCN(CC)C=1C1CCCCC1)ccc3C	7.7
78	O(C)c1cc(c2[nH]c3[NH+]=C(N(CCc3c2c1C)CC)C1CCCCC1)C	7.6
79	O(C)c1cc2c([nH]c3[NH+]=C(N(CCc23)CC)C2CCCCC2)c(C)c1C	7.6
80	Clc1c(c2[nH]c3[NH+]=C(N(CCc3c2cc1OC)CC)C1CCCCC1)C	7.6
81	Clc1cc(OC)c(c2c1[nH]c1[NH+]=C(N(CCc12)CC)C1CCCCC1)C	7.6
82	O1c2c(OC1)c1[nH]c3[NH+]=C(N(CCc3c1cc2OC)CC)C1CCCCC1	7.6
83	O(CC)c1cc(c2[nH]c3[NH+]=C(N(CCc3c2c1)CC)C1CCCCC1)C	7.6
84	S(C)c1cc(c2[nH]c3[NH+]=C(N(CCc3c2c1)CC)C1CCCCC1)C	7.6
85	ClC(F)(F)Oc1cc2c3CCN(CC)C(=[NH+]c3[nH]c2cc1)C1CCCCC1	7.6
86	O(C)c1c2c(c3[nH]c4[NH+]=C(N(CCc4c3c1)CC)C1CCCCC1)cccc2	7.6
87	s1c2c3CCN(CC)C(=[NH+]c3[nH]c2cc1)C1CCCCC1	7.6
88	Clc1c(OC)c2[nH]c3[NH+]=C(N(CCc3c2cc1OC)CC)C1CCCCC1	7.6
89	Fc1cc2[nH]c3[NH+]=C(N(CCc3c2cc1OC(F)(F)F)CC)C1CCCCC1	7.6
90	O(C)c1c(C)c(c2[nH]c3[NH+]=C(N(CCc3c2c1C)CC)C1CCCCC1)C	7.6
91	Fc1cc2[nH]c3[NH+]=C(N(CCc3c2cc1OCCC)CC)C1CCCCC1	7.6
92	Clc1cc2c3CCN(CC)C(=[NH+]c3[nH]c2cc1F)C1CCCCC1	7.6
93	O(C)c1c2CCCc2c2[nH]c3[NH+]=C(N(CCc3c2c1)CC)C1CCCCC1	7.6
94	O1CCc2c1cc1c([nH]c3[NH+]=C(N(CCc13)CC)C1CCCCC1)c2C	7.6
95	FC(F)Oc1cc2[nH]c3[NH+]=C(N(CCc3c2cc1OC)CC)C1CCCCC1	7.6
96	s1c2c3CCN(CC)C(=[NH+]c3[nH]c2cc1C)C1CCCCC1	7.6
97	s1c2c(cc3[nH]c4[NH+]=C(N(CCc4c3c2)CC)C2CCCCC2)cc1	7.6
98	O(CCC)c1cc(c2[nH]c3[NH+]=C(N(CCc3c2c1)CC)C1CCCCC1)C	7.6
99	Fc1c2c3CCN(CC)C(=[NH+]c3[nH]c2cc(F)c1C)C1CCCCC1	7.6
100	Clc1cc(OCCF)cc2c1[nH]c1[NH+]=C(N(CCc12)CC)C1CCCCC1	7.6
101	O(C(C)C)c1cc2c3CCN(CC)C(=[NH+]c3[nH]c2cc1C)C1CCCCC1	7.6
102	O(CC1CC1)c1cc(c2[nH]c3[NH+]=C(N(CCc3c2c1)CC)C1CCCCC1)C	7.6
103	O(CCC)c1cc(c2[nH]c3[NH+]=C(N(CCc3c2c1C)CC)C1CCCCC1)C	7.6
104	Clc1cc(SC)c2[nH]c3[NH+]=C(N(CCc3c2c1)CC)C1CCCCC1	7.6
105	O(C)c1c2c(c3[nH]c4[NH+]=C(N(CCc4c3c1C)CC)C1CCCCC1)cccc2	7.6
106	O(CCC)c1cc2c([nH]c3[NH+]=C(N(CCc23)CC)C2CCCCC2)c(C)c1C	7.6
107	O(CC)c1c2c(c3[nH]c4[NH+]=C(N(CCc4c3c1)CC)C1CCCCC1)cccc2	7.6
108	O(C)c1cc2c3CCN(CC)C(=[NH+]c3[nH]c2cc1CCC)C1CCCCC1	7.6

109	Clc1sc2c3CCN(CC)C(=[NH+]c3[nH]c2c1)C1CCCCC1	7.6
110	Fc1cc(F)cc2[nH]c3[NH+]=C(N(CCc3c12)CC)C1CCCCC1	7.6
111	O(C)c1ccc2[nH]c3[NH+]=C(N(CCc3c2c1C#N)CC)C1CCCCC1	7.6
112	Fc1cc(cc2[nH]c3[NH+]=C(N(CCc3c12)CC)C1CCCCC1)C	7.6
113	s1c2c3CCN(CC)C(=[NH+]c3[nH]c2cc1C(C)C)C1CCCCC1	7.6
114	[NH+]=1C=2Nc3n(C=2CCN(CC)C=1C1CCCCC1)c(cc3)CCC	7.6
115	S(C)c1cc(SC)cc2c1[nH]c1[NH+]=C(N(CCc12)CC)C1CCCCC1	7.6
116	Fc1cc2[nH]c3[NH+]=C(N(CCc3c2cc1OC)CC)C1CCCCC1	7.5
117	[NH+]=1c2[nH]c3c(cc(cc3)CC)c2CCN(CC)C=1C1CCCCC1	7.5
118	FC(F)Oc1cc2c3CCN(CC)C(=[NH+]c3[nH]c2cc1)C1CCCCC1	7.5
119	O(C(C)C)c1cc2c3CCN(CC)C(=[NH+]c3[nH]c2cc1)C1CCCCC1	7.5
120	[NH+]=1c2[nH]c3c(cc(cc3)C)c2CCN(CC)C=1C1CCCCC1	7.5
121	[NH+]=1c2[nH]c3c(c2CCN(CC)C=1C1CCCCC1)cccc3	7.5
122	Fc1cc2c3CCN(CC)C(=[NH+]c3[nH]c2cc1)C1CCCCC1	7.5
123	Fc1cc2[nH]c3[NH+]=C(N(CCc3c2cc1OC(F)F)CC)C1CCCCC1	7.5
124	O(C)c1cc(OC)c2[nH]c3[NH+]=C(N(CCc3c2c1C)CC)C1CCCCC1	7.5
125	O(CC=C)c1cc2c3CCN(CC)C(=[NH+]c3[nH]c2cc1)C1CCCCC1	7.5
126	O1c2c(OC1)c1[nH]c3[NH+]=C(N(CCc3c1c(C)c2OC)CC)C1CCCCC1	7.5
127	O(COC)c1cc2c3CCN(CC)C(=[NH+]c3[nH]c2cc1)C1CCCCC1	7.5
128	S(C)c1cc2c3CCN(CC)C(=[NH+]c3[nH]c2cc1C)C1CCCCC1	7.5
129	Fc1cc2[nH]c3[NH+]=C(N(CCc3c2cc1OC(C)C)CC)C1CCCCC1	7.5
130	Clc1cc2[nH]c3[NH+]=C(N(CCc3c2cc1OCC)CC)C1CCCCC1	7.5
131	O1CCOc2c1cc1c3CCN(CC)C(=[NH+]c3[nH]c1c2)C1CCCCC1	7.5
132	Clc1cc2[nH]c3[NH+]=C(N(CCc3c2cc1S)CC)C1CCCCC1	7.5
133	O(C)c1cc(c2[nH]c3[NH+]=C(N(CCc3c2c1OC)CC)C1CCCCC1)C	7.5
134	O(CC)c1cc2c3CCN(CC)C(=[NH+]c3[nH]c2cc1CC)C1CCCCC1	7.5
135	[NH+]=1c2[nH]c3cc(C)c(cc3c2CCN(CC)C=1C1CCCCC1)C	7.5
136	Clc1cc2[nH]c3[NH+]=C(N(CCc3c2cc1OCC#C)CC)C1CCCCC1	7.5
137	Fc1cc2c3CCN(CC)C(=[NH+]c3[nH]c2cc1C)C1CCCCC1	7.5
138	[NH+]=1c2[nH]c3c(cc(cc3)\C=C\C)c2CCN(CC)C=1C1CCCCC1	7.5
139	Sc1cc2[nH]c3[NH+]=C(N(CCc3c2cc1F)CC)C1CCCCC1	7.5
140	O(CC#C)c1cc2c3CCN(CC)C(=[NH+]c3[nH]c2cc1)C1CCCCC1	7.5
141	Fc1c2c3CCN(CC)C(=[NH+]c3[nH]c2ccc1OC(F)(F)F)C1CCCCC1	7.5
142	Clc1c2c3CCN(CC)C(=[NH+]c3[nH]c2ccc1CC)C1CCCCC1	7.5
143	[NH+]=1c2[nH]c3c(c2CCN(CC)C=1C1CCCCC1)c(ccc3)C[NH+]=[CH-]	7.5
144	Sc1c2c3CCN(CC)C(=[NH+]c3[nH]c2ccc1)C1CCCCC1	7.5
145	Clc1c2c3CCN(CC)C(=[NH+]c3[nH]c2ccc1OCCC)C1CCCCC1	7.5
146	Fc1cc2c3CCN(CC)C(=[NH+]c3[nH]c2cc1F)C1CCCCC1	7.5
147	O(C(OC)=O)c1cc2c3CCN(CC)C(=[NH+]c3[nH]c2cc1)C1CCCCC1	7.5
148	[NH+]=1c2[nH]c3CCC=Cc3c2CCN(CC)C=1C1CCCCC1	7.5
149	O(N(C)C)c1cc(c2[nH]c3[NH+]=C(N(CCc3c2c1)CC)C1CCCCC1)C	7.5
150	O(C)c1c(C)c(OC)c2[nH]c3[NH+]=C(N(CCc3c2c1C)CC)C1CCCCC1	7.5
151	Brc1sc2c3CCN(CC)C(=[NH+]c3[nH]c2c1)C1CCCCC1	7.5
152	o1c2c3c4CCN(CC)C(=[NH+]c4[nH]c3ccc2cc1)C1CCCCC1	7.5
153	FCc1c2c3CCN(CC)C(=[NH+]c3[nH]c2ccc1)C1CCCCC1	7.5
154	O(CC1CC1)c1cc2c3CCN(CC)C(=[NH+]c3[nH]c2cc1)C1CCCCC1	7.5
155	Brc1cc2[nH]c3[NH+]=C(N(CCc3c2cc1)CC)C1CCCCC1	7.5
156	[NH+]=1c2[nH]c3cc(C)c(cc3c2CCN(CC)C=1C1CCCCC1)CC	7.5
157	Brc1cc2n(NC=3[NH+]=C(N(CCC2=3)CC)C2CCCCC2)c1	7.5

158	[NH+]=1c2[nH]c3cc(C)c(N(C)C)cc3c2CCN(CC)C=1C1CCCCC1	7.5
159	Clc1cc2c3CCN(CC)C(=[NH+]c3[nH]c2cc1Cl)C1CCCCC1	7.5
160	[NH+]=1C=2Nn3cc(cc3C=2CCN(CC)C=1C1CCCCC1)C	7.5
161	O(CC(C)C)c1cc2c3CCN(CC)C(=[NH+]c3[nH]c2cc1)C1CCCCC1	7.5
162	[NH+]=1c2[nH]c3c(cc(cc3)C(C)C)c2CCN(CC)C=1C1CCCCC1	7.5
163	Clc1c2c3CCN(CC)C(=[NH+]c3[nH]c2ccc1)C1CCCCC1	7.5
164	[NH+]=1c2[nH]c3C=Cn4c(-c3c2CCN(CC)C=1C1CCCCC1)ccc4	7.5
165	s1c2c3CCN(CC)C(=[NH+]c3[nH]c2cc1CC)C1CCCCC1	7.5
166	O1c2c(CCC1(C)C)c1c3CCN(CC)C(=[NH+]c3[nH]c1cc2)C1CCCCC1	7.5
167	[NH+]=1c2[nH]c3c(c2CCN(CC)C=1C1CCCCC1)cncc3	7.5
168	Brc1cc2[nH]c3[NH+]=C(N(CCc3c2cc1C)CC)C1CCCCC1	7.5
169	Clc1c(c2c3CCN(CC)C(=[NH+]c3[nH]c2cc1C)C1CCCCC1)C	7.5
170	o1c2c(cc1C)cc1[nH]c3[NH+]=C(N(CCc3c1c2)CC)C1CCCCC1	7.5
171	O(CCC)c1cc2c3CCN(CC)C(=[NH+]c3[nH]c2cc1C)C1CCCCC1	7.5
172	s1ncc2c1c1c3CCN(CC)C(=[NH+]c3[nH]c1cc2)C1CCCCC1	7.5
173	S(C(F)F)c1cc2c3CCN(CC)C(=[NH+]c3[nH]c2cc1)C1CCCCC1	7.5
174	SCc1cc2[nH]c3[NH+]=C(N(CCc3c2cc1)CC)C1CCCCC1	7.5
175	FC(F)(F)c1cc2c3CCN(CC)C(=[NH+]c3[nH]c2cc1)C1CCCCC1	7.5
176	[NH+]=1c2[nH]c3c(c2CCN(CC)C=1C1CCCCC1)cc1CCCc1c3	7.5
177	O(Cc1c2c3CCN(CC)C(=[NH+]c3[nH]c2ccc1)C1CCCCC1)C	7.5
178	[NH+]=1c2[nH]c3cc(C)c(cc3c2CCN(CC)C=1C1CCCCC1)C(C)C	7.5
179	Clc1cc2c([nH]c3[NH+]=C(N(CCc23)CC)C2CCCCC2)c(OC)c1C	7.5
180	O(C)c1cc2c3CCN(CC)C(=[NH+]c3[nH]c2cc1C)C1CCCCC1	7.4
181	Clc1cc2[nH]c3[NH+]=C(N(CCc3c2cc1OC)CC)C1CCCCC1	7.4
182	Sc1cc2c3CCN(CC)C(=[NH+]c3[nH]c2cc1)C1CCCCC1	7.4
183	[NH+]=1c2[nH]c3c(cc(cc3)C#C)c2CCN(CC)C=1C1CCCCC1	7.4
184	Clc1cc2c3CCN(CC)C(=[NH+]c3[nH]c2cc1)C1CCCCC1	7.4
185	S(C)c1cc2c3CCN(CC)C(=[NH+]c3[nH]c2cc1)C1CCCCC1	7.4
186	Fc1c2c3CCN(CC)C(=[NH+]c3[nH]c2ccc1OCC)C1CCCCC1	7.4
187	Fc1c2c3CCN(CC)C(=[NH+]c3[nH]c2ccc1OC(F)F)C1CCCCC1	7.4
188	O(C1CC1)c1cc2c3CCN(CC)C(=[NH+]c3[nH]c2cc1)C1CCCCC1	7.4
189	Clc1c2c3CCN(CC)C(=[NH+]c3[nH]c2ccc1OC)C1CCCCC1	7.4
190	FC(F)(F)Oc1cc2c3CCN(CC)C(=[NH+]c3[nH]c2cc1)C1CCCCC1	7.4
191	O(C1CCC1)c1cc2c3CCN(CC)C(=[NH+]c3[nH]c2cc1)C1CCCCC1	7.4
192	FCCOc1cc2c3CCN(CC)C(=[NH+]c3[nH]c2cc1)C1CCCCC1	7.4
193	[NH+]=1c2[nH]c3c(cc(cc3)C=C)c2CCN(CC)C=1C1CCCCC1	7.4
194	O(CC)c1c(c2c3CCN(CC)C(=[NH+]c3[nH]c2cc1C)C1CCCCC1)C	7.4
195	O(CCC)c1cc2c3CCN(CC)C(=[NH+]c3[nH]c2cc1)C1CCCCC1	7.4
196	s1c2c3CCN(CC)C(=[NH+]c3[nH]c2cc1CSC)C1CCCCC1	7.4
197	O(C(C)C)c1ccc2[nH]c3[NH+]=C(N(CCc3c2c1C)CC)C1CCCCC1	7.4
198	Clc1cc2[nH]c3[NH+]=C(N(CCc3c2cc1OC#CC)CC)C1CCCCC1	7.4
199	Clc1cc2c3CCN(CC)C(=[NH+]c3[nH]c2cc1C)C1CCCCC1	7.4
200	Clc1cc2[nH]c3[NH+]=C(N(CCc3c2cc1OC(F)F)CC)C1CCCCC1	7.4
201	Clc1ccc2[nH]c3[NH+]=C(N(CCc3c2c1F)CC)C1CCCCC1	7.4
202	O1n2c(C=C1)c1c3CCN(CC)C(=[NH+]c3[nH]c1c2)C1CCCCC1	7.4
203	O1CCCc2c3c4CCN(CC)C(=[NH+]c4[nH]c3ccc12)C1CCCCC1	7.4
204	Clc1ccc2[nH]c3[NH+]=C(N(CCc3c2c1C)CC)C1CCCCC1	7.4
205	S1c2c3c4CCN(CC)C(=[NH+]c4[nH]c3ccc2OC1)C1CCCCC1	7.4
206	Fc1c2c3CCN(CC)C(=[NH+]c3[nH]c2ccc1)C1CCCCC1	7.4

207	Clc1cc2[nH]c3[NH+]=C(N(CCc3c2cc1OCCC)CC)C1CCCCC1	7.4
208	Fc1c2c3CCN(CC)C(=[NH+]c3[nH]c2ccc1C)C1CCCCC1	7.4
209	Fc1c2c3CCN(CC)C(=[NH+]c3[nH]c2ccc1N(C)C)C1CCCCC1	7.4
210	O1CCOc2c1c1c3CCN(CC)C(=[NH+]c3[nH]c1cc2)C1CCCCC1	7.4
211	[NH+]=1c2[nH]c3c(cc(N(C)C)cc3)c2CCN(CC)C=1C1CCCCC1	7.4
212	O(CC)c1ccc2[nH]c3[NH+]=C(N(CCc3c2c1C)CC)C1CCCCC1	7.4
213	Clc1c2c3CCN(CC)C(=[NH+]c3[nH]c2ccc1OC(C)C)C1CCCCC1	7.4
214	s1c2c(CCOC2)c2c3CCN(CC)C(=[NH+]c3[nH]c12)C1CCCCC1	7.4
215	Clc1cc2[nH]c3[NH+]=C(N(CCc3c2cc1OC(C)C)CC)C1CCCCC1	7.4
216	Fc1c2c3CCN(CC)C(=[NH+]c3[nH]c2ccc1F)C1CCCCC1	7.4
217	S(=O)(=O)(C)c1cc2c3CCN(CC)C(=[NH+]c3[nH]c2nc1)C1CCCCC1	7.4
218	O1c2c(OC1)cc1[nH]c3[NH+]=C(N(CCc3c1c2)CC)C1CCCCC1	7.4
219	Clc1ccc2[nH]c3[NH+]=C(N(CCc3c2c1CC)CC)C1CCCCC1	7.4
220	Clc1cc2[nH]c3[NH+]=C(N(CCc3c2cc1OCC=C)CC)C1CCCCC1	7.4
221	O1c2c(CCCC1)c1c3CCN(CC)C(=[NH+]c3[nH]c1cc2C)C1CCCCC1	7.4
222	[NH+]=1C=2Nn3c(C=2CCN(CC)C=1C1CCCCC1)ccc3	7.4
223	O(C)c1c2c3CCN(CC)C(=[NH+]c3[nH]c2ccc1)C1CCCCC1	7.4
224	o1c2c(cc3[nH]c4[NH+]=C(N(CCc4c3c2)CC)C2CCCCC2)cc1	7.4
225	O(C)c1cc2c3CCN(CC)C(=[NH+]c3[nH]c2cc1[C@H](CC)C)C1CCCCC1	7.4
226	Brc1ccc2[nH]c3[NH+]=C(N(CCc3c2c1F)CC)C1CCCCC1	7.4
227	FC(F)(F)Oc1ccc2[nH]c3[NH+]=C(N(CCc3c2c1C)CC)C1CCCCC1	7.4
228	Fc1ccc2[nH]c3[NH+]=C(N(CCc3c2c1OC)CC)C1CCCCC1	7.4
229	FC(F)c1c2c3CCN(CC)C(=[NH+]c3[nH]c2ccc1)C1CCCCC1	7.4
230	[NH+]=1c2[nH]c3c(c2CCN(CC)C=1C1CCCCC1)c(C)c(N(C)C)cc3	7.4
231	FCCCc1cc2c3CCN(CC)C(=[NH+]c3[nH]c2cc1)C1CCCCC1	7.4
232	s1ncc2c1cc1[nH]c3[NH+]=C(N(CCc3c1c2)CC)C1CCCCC1	7.4
233	Clc1c2c3CCN(CC)C(=[NH+]c3[nH]c2ccc1C)C1CCCCC1	7.4
234	Brc1c2c3CCN(CC)C(=[NH+]c3[nH]c2ccc1)C1CCCCC1	7.4
235	O(CC)c1c2c3CCN(CC)C(=[NH+]c3[nH]c2ccc1)C1CCCCC1	7.4
236	S(Cc1c2c3CCN(CC)C(=[NH+]c3[nH]c2ccc1)C1CCCCC1)C	7.4
237	o1nnc2c1c1c3CCN(CC)C(=[NH+]c3[nH]c1cc2)C1CCCCC1	7.4
238	O(C1CCCCC1)c1cc2c3CCN(CC)C(=[NH+]c3[nH]c2cc1)C1CCCCC1	7.4
239	O(C)c1cc2c3CCN(CC)C(=[NH+]c3[nH]c2cc1)C1CCCCC1	7.3
240	O(C)c1ccc2[nH]c3[NH+]=C(N(CCc3c2c1C)CC)C1CCCCC1	7.3
241	O(C)c1cc2c3CCN(CC)C(=[NH+]c3[nH]c2cc1OC)C1CCCCC1	7.3
242	O(C)c1c(c2c3CCN(CC)C(=[NH+]c3[nH]c2cc1C)C1CCCCC1)C	7.3
243	Fc1c2c3CCN(CC)C(=[NH+]c3[nH]c2ccc1OC)C1CCCCC1	7.3
244	O(CC)c1cc2c3CCN(CC)C(=[NH+]c3[nH]c2cc1)C1CCCCC1	7.3
245	O1CCCOc2c1cc1c3CCN(CC)C(=[NH+]c3[nH]c1c2)C1CCCCC1	7.3
246	O(C)c1c(c2c3CCN(CC)C(=[NH+]c3[nH]c2cc1OC)C1CCCCC1)C	7.3
247	S1c2c(OC1)cc1c3CCN(CC)C(=[NH+]c3[nH]c1c2)C1CCCCC1	7.3
248	Fc1c2c3CCN(CC)C(=[NH+]c3[nH]c2cc(F)c1OC)C1CCCCC1	7.3
249	O(C(C)(C)C)c1cc2c3CCN(CC)C(=[NH+]c3[nH]c2cc1)C1CCCCC1	7.3
250	S(=O)(=O)(C)c1cc(c2[nH]c3[NH+]=C(N(CCc3c2c1)CC)C1CCCCC1)C	7.3
251	[NH+]=1c2[nH]c3cc(ccc3c2CCN(CC)C=1C1CCCCC1)C[NH+]=[CH-]	7.3
252	Clc1c2c3CCN(CC)C(=[NH+]c3[nH]c2cc(OC)c1OC)C1CCCCC1	7.3
253	O(CC)c1c2c3CCN(CC)C(=[NH+]c3[nH]c2ccc1OC)C1CCCCC1	7.3
254	O1Cc2c(C1)cc1[nH]c3[NH+]=C(N(CCc3c1c2)CC)C1CCCCC1	7.3
255	O([C@H](CC)C)c1cc2c3CCN(CC)C(=[NH+]c3[nH]c2cc1)C1CCCCC1	7.3

256	Clc1ccc2[nH]c3[NH+]=C(N(CCc3c2c1OC)CC)C1CCCCC1	7.3
257	s1c2c(c3[nH]c4[NH+]=C(N(CCc4c13)CC)C1CCCCC1)cccc2OC	7.3
258	O1CCC[C@H]1c1cc2c3CCN(CC)C(=[NH+]c3[nH]c2cc1)C1CCCCC1	7.3
259	Clc1cc2c3CCN(CC)C(=[NH+]c3[nH]c2cc1CC)C1CCCCC1	7.3
260	O(C(C)C)c1cc2[nH]c3[NH+]=C(N(CCc3c2cc1OC)CC)C1CCCCC1	7.3
261	S(C)c1cc2[nH]c3[NH+]=C(N(CCc3c2cc1OC)CC)C1CCCCC1	7.3
262	[NH+]=1c2[nH]c3c(c2CCN(CC)C=1C1CCCCC1)cc1N(CCc1c3)C	7.3
263	S1Cc2c3CCN(CC)C(=[NH+]c3[nH]c2C1)C1CCCCC1	7.3
264	O(CCC)c1ccc2[nH]c3[NH+]=C(N(CCc3c2c1C)CC)C1CCCCC1	7.3
265	[NH+]=1c2[nH]c3c(cc(-n4nccc4C)cc3)c2CCN(CC)C=1C1CCCCC1	7.3
266	[NH+]=1c2[nH]c3CC=CCc3c2CCN(CC)C=1C1CCCCC1	7.3
267	Fc1c(cc2[nH]c3[NH+]=C(N(CCc3c2c1F)CC)C1CCCCC1)C	7.3
268	Clc1c2c3CCN(CC)C(=[NH+]c3[nH]c2ccc1Cl)C1CCCCC1	7.3
269	FC(F)(F)COc1cc2c3CCN(CC)C(=[NH+]c3[nH]c2cc1)C1CCCCC1	7.3
270	[NH+]=1c2[nH]c3cc(ccc3c2CCN(CC)C=1C1CCCCC1)CC=C	7.3
271	S(=O)(C)c1cc2c3CCN(CC)C(=[NH+]c3[nH]c2cc1)C1CCCCC1	7.2
272	O(C)c1cc2c3CCN(CC)C(=[NH+]c3[nH]c2cc1CC)C1CCCCC1	7.2
273	Sc1cc2c3CCN(CC)C(=[NH+]c3[nH]c2cc1C)C1CCCCC1	7.2
274	O(CC)c1cc2[nH]c3[NH+]=C(N(CCc3c2cc1OC)CC)C1CCCCC1	7.2
275	FC(F)(C)c1cc2c3CCN(CC)C(=[NH+]c3[nH]c2cc1)C1CCCCC1	7.2
276	o1c2[nH]c3[NH+]=C(N(CCc3c2cc1COC)CC)C1CCCCC1	7.2
277	O1CCCc2c1cc1c3CCN(CC)C(=[NH+]c3[nH]c1c2)C1CCCCC1	7.2
278	FCCc1cc2[nH]c3[NH+]=C(N(CCc3c2cc1OC)CC)C1CCCCC1	7.2
279	O1CCc2c(C1)ccc1[nH]c3[NH+]=C(N(CCc3c12)CC)C1CCCCC1	7.2
280	S(=O)(=O)(C)c1cc2c3CCN(CC)C(=[NH+]c3[nH]c2cc1C)C1CCCCC1	7.2
281	s1c2c3CCN(CC)C(=[NH+]c3[nH]c2cc1S(=O)(=O)CC)C1CCCCC1	7.2
282	O1CCc2[nH]c3[NH+]=C(N(CCc3c2C1)CC)C1CCCCC1	7.2
283	Clc1c2c3CCN(CC)C(=[NH+]c3[nH]c2ccc1OCC)C1CCCCC1	7.2
284	O1CCc2c1cc1c3CCN(CC)C(=[NH+]c3[nH]c1c2)C1CCCCC1	7.2
285	O(\N=C\c1cc2c3CCN(CC)C(=[NH+]c3[nH]c2cc1)C1CCCCC1)C	7.2
286	O1CCc2n3C=4CCN(CC)C(=[NH+]C=4Nc3cc2C1)C1CCCCC1	7.2
287	O([C@H](C#N)C)c1cc2c3CCN(CC)C(=[NH+]c3[nH]c2cc1)C1CCCCC1	7.2
288	O(C)c1cc2c3CCN(CC)C(=[NH+]c3[nH]c2cc1N(C)C)C1CCCCC1	7.2
289	O(C)c1c(OC)c2c3CCN(CC)C(=[NH+]c3[nH]c2cc1OC)C1CCCCC1	7.2
290	s1c2c(c3c4CCN(CC)C(=[NH+]c4[nH]c13)C1CCCCC1)[C@@H](OC2)C	7.2
291	S1CCc2[nH]c3[NH+]=C(N(CCc3c12)CC)C1CCCCC1	7.2
292	s1c2c3CCN(CC)C(=[NH+]c3[nH]c2cc1SC)C1CCCCC1	7.2
293	Clc1cc2[nH]c3[NH+]=C(N(CCc3c2c(OC)c1OC)CC)C1CCCCC1	7.2
294	Clc1cc2[nH]c3[NH+]=C(N(CCc3c2cc1C(=O)C)CC)C1CCCCC1	7.2
295	O(c1cc2c3CCN(CC)C(=[NH+]c3[nH]c2cc1)C1CCCCC1)c1ncccn1	7.2
296	O1CCN(c2c1cc1c3CCN(CC)C(=[NH+]c3[nH]c1c2)C1CCCCC1)C	7.2
297	O(Cc1cc2n(NC=3[NH+]=C(N(CCC2=3)CC)C2CCCCC2)c1)C	7.1
298	O=C(C)c1ccc2[nH]c3[NH+]=C(N(CCc3c2c1C)CC)C1CCCCC1	7.1
299	FC(F)COc1cc2c3CCN(CC)C(=[NH+]c3[nH]c2cc1)C1CCCCC1	7.1
300	O(CC(C)=C)c1cc2c3CCN(CC)C(=[NH+]c3[nH]c2cc1)C1CCCCC1	7.1
301	s1c2[nH]c3[NH+]=C(N(CCc3c2cc1OC)CC)C1CCCCC1	7.1
302	O1C[C@@H]1COc1cc2c3CCN(CC)C(=[NH+]c3[nH]c2cc1)C1CCCCC1	7.1
303	O(C)c1c2c3CCN(CC)C(=[NH+]c3[nH]c2ccc1OC)C1CCCCC1	7.1
304	O=C(C)c1c(c2c3CCN(CC)C(=[NH+]c3[nH]c2cc1C)C1CCCCC1)C	7.1

305	O(C(=O)C)c1cc2[nH]c3[NH+]=C(N(CCc3c2cc1OC)CC)C1CCCCC1	7.1
306	s1nc(c2c3CCN(CC)C(=[NH+]c3[nH]c12)C1CCCCC1)COC	7.1
307	O=C(N1CCC1)c1cc2c3CCN(CC)C(=[NH+]c3[nH]c2cc1)C1CCCCC1	7.1
308	[NH+]=1c2[nH]c3c(cc(cc3)C3(CC3)C#N)c2CCN(CC)C=1C1CCCCC1	7.1
309	O(C)c1cc2c3CCN(CC)C(=[NH+]c3[nH]c2cc1CC#N)C1CCCCC1	7.0
310	O1C[C@H]1c1cc2[nH]c3[NH+]=C(N(CCc3c2cc1)CC)C1CCCCC1	7.0
311	Fc1c2c3CCN(CC)C(=[NH+]c3[nH]c2ccc1C(=O)C)C1CCCCC1	7.0
312	S1N2C(OC=C1)=CN1C=3CCN(CC)C(=[NH+]C=3NC1=C2)C1CCCCC1	7.0
313	S(=O)(=O)(C)c1cc2c3CCN(CC)C(=[NH+]c3[nH]c2cc1F)C1CCCCC1	6.9
314	O(CC#N)c1cc2c3CCN(CC)C(=[NH+]c3[nH]c2cc1)C1CCCCC1	6.9
315	O=C(Cc1cc2n(C=3CCN(CC)C(=[NH+]C=3N2)C2CCCCC2)c1C)C	6.9
316	S(=O)(=O)(C)c1cc2c3CCN(CC)C(=[NH+]c3[nH]c2cc1)C1CCCCC1	6.8
317	S(=O)(C)c1cc2n(c1)C=1CCN(CC)C(=[NH+]C=1N2)C1CCCCC1	6.8
318	S(=O)(=O)(CC)c1cc2c3CCN(CC)C(=[NH+]c3[nH]c2cc1)C1CCCCC1	6.7
319	O(CC=O)c1cc2c3CCN(CC)C(=[NH+]c3[nH]c2cc1)C1CCCCC1	6.7

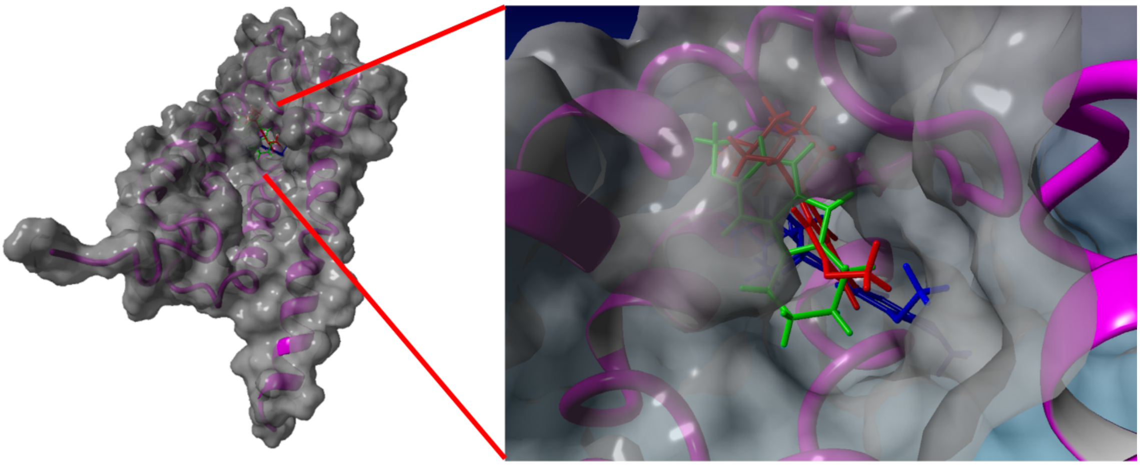

Figure S3. Comparison of the best docked poses for ibogaine (red), pinoline (green), and compound 2_4 (blue).

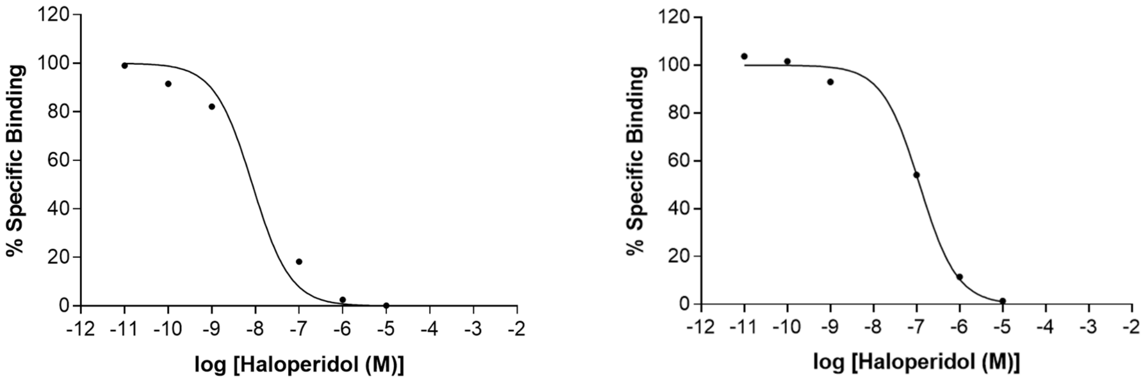

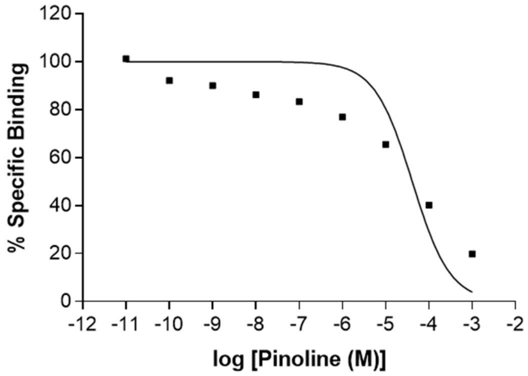

Figure S4. Binding curves. Haloperidol on σ_1 K_i = 3.2 nM (up-left) and σ_2 K_i = 102.2 nM (up-right) receptor. Pinoline on σ_2 receptor K_i = 35.4 µM (down); K_i for pinoline at σ_1 receptor >100 µM.
